# An Explainable Radiomics-Based Classification Model for Sarcoma Diagnosis

**DOI:** 10.3390/diagnostics15162098

**Published:** 2025-08-20

**Authors:** Simona Correra, Arnar Evgení Gunnarsson, Marco Recenti, Francesco Mercaldo, Vittoria Nardone, Antonella Santone, Halldór Jónsson, Paolo Gargiulo

**Affiliations:** 1 Department of Medicine and Health Sciences “Vincenzo Tiberio”, University of Molise, 86100 Campobasso, Italy; s.correra@studenti.unimol.it (S.C.); francesco.mercaldo@unimol.it (F.M.); vittoria.nardone@unimol.it (V.N.); antonella.santone@unimol.it (A.S.); 2 Institute of Biomedical and Neural Engineering, Reykjavik University, 102 Reykjavik, Iceland; arnareg@ru.is (A.E.G.); marcor@ru.is (M.R.); halldorjon@ru.is (H.J.J.); 3 Department of Science, Landspitali University Hospital, 105 Reykjavik, Iceland

**Keywords:** sarcoma diagnosis, radiomics, machine learning, classification, explainability

## Abstract

**Objective**: This study introduces an explainable, radiomics-based machine learning framework for the automated classification of sarcoma tumors using MRI. The approach aims to empower clinicians, reducing dependence on subjective image interpretation. **Methods**: A total of 186 MRI scans from 86 patients diagnosed with bone and soft tissue sarcoma were manually segmented to isolate tumor regions and corresponding healthy tissue. From these segmentations, 851 handcrafted radiomic features were extracted, including wavelet-transformed descriptors. A Random Forest classifier was trained to distinguish between tumor and healthy tissue, with hyperparameter tuning performed through nested cross-validation. To ensure transparency and interpretability, model behavior was explored through Feature Importance analysis and Local Interpretable Model-agnostic Explanations (LIME). **Results**: The model achieved an F1-score of 0.742, with an accuracy of 0.724 on the test set. LIME analysis revealed that texture and wavelet-based features were the most influential in driving the model’s predictions. **Conclusions**: By enabling accurate and interpretable classification of sarcomas in MRI, the proposed method provides a non-invasive approach to tumor classification, supporting an earlier, more personalized and precision-driven diagnosis. This study highlights the potential of explainable AI to assist in more secure clinical decision-making.

## 1. Introduction

Sarcomas are a rare and diverse group of malignant tumors arising from mesenchymal tissue [1,2]. In the United States, sarcomas account for 1% of new cancer cases and cancer-related deaths [3]. But this statistic hides a sad truth: among children and adolescents, sarcomas strike with disproportionate force, responsible for 13% of all cancer-related deaths in patients between 0 and 19 years [4]. Several studies have reported a higher incidence of sarcoma in males and it is particularly evident in childhood sarcomas, which show a stronger association with the male sex. However, the biological mechanisms underlying these sex differences remain poorly understood [5].

Clinical signs are often nonspecific, which frequently leads to diagnostic delays and negatively impacts patient outcomes [6]. Moreover, since soft tissue sarcoma is a rare tumor, general practitioners typically diagnose only one case in 24 years of practice. Their histological and molecular heterogeneity, combined with low incidence and aggressive clinical behavior, pose significant challenges for timely and accurate diagnosis and treatment [2]. Early diagnosis improves outcomes, as it can prevent the growth of the mass and decrease the risk of metastases, which are predictors of survival [6]. As a matter of fact, Pisters et al. reported that patients with soft tissue sarcomas larger than 5 cm had a relative risk of death 2.1 times higher than those with tumors smaller than 5 cm [7]. Then, Kolovich et al. reported that each additional centimeter in tumor size leads to a 3–5% reduction in the survival rate.

Radiological imaging plays a crucial role in the diagnostic process. For bone sarcomas, radiography, MRI, and CT scans are the primary diagnostic tools. Instead, ultrasound and MRI are the main modalities used to evaluate soft-tissue lesions [8,9]. However, their interpretation remains subjective, as it relies on the radiologist’s expertise [10].

Although histopathology remains the gold standard for definitive diagnosis, it is still obtained after an invasive procedure and therefore not without limitations. To address these challenges, genetic profiling has emerged as a valuable complementary approach, identifying subtype-specific mutations and molecular signatures that enhance classification accuracy and support targeted therapeutic strategies [11,12].

Nowadays, Artificial intelligence (AI) offers a helping hand to physicians to address these limitations [13]. Through the extraction and analysis of quantitative features, AI algorithms can identify nuanced patterns imperceptible to human observers, facilitate standardized diagnostic workflows, and potentially enhance diagnostic accuracy [14,15,16].

This study evaluates the possibility of using machine learning, in particular Random Forest (RF), to distinguish healthy tissue from sarcomas across a broad spectrum of anatomic sites, MRI protocols, and different acquisition image conditions. Radiomic features were extracted from manually segmented MR images and used to train a RF classifier. Using a heterogeneous, real-world dataset collected between 2004 and 2023, we assess the robustness and generalizability of the proposed approach. Our broader aim is to demonstrate how AI-driven image analysis can serve as a second opinion alongside traditional diagnostic workflows, helping to reduce inter-observer variability and ultimately contributing to more consistent and effective patient management through a more tailored diagnostic process.

The following section describes the Materials and Methods. Section 3 reports the results obtained, followed by Section 4, which includes a review of the state of the art. Finally, the Conclusion section summarizes the main findings.

## 2. Materials and Methods

### 2.1. Dataset

#### 2.1.1. Sarcoma Cases

To develop and evaluate the proposed method, MRI scans from the IceSG were used. The IceSG (Icelandic Sarcoma Group) dataset consists of sarcoma cases collected by Landspítali University Hospital. The patient data were approved by the Landspítali Ethics Committee (no. 5), dated 6 February 2024, while the study received approval from Landspítali Scientific Research Committee (No. 93), dated 9 January 2024. The dataset comprises clinical information on patients diagnosed with various bone and soft tissue tumors, with diagnostic examinations performed over a 20-year period, from 2004 to 2023. The average age at diagnosis is approximately 48.78 (±23.52) years, reflecting a wide age distribution, with patient ages ranging from a minimum of 9 years to a maximum of 108 years.

The majority of cases are classified as soft tissue sarcomas, accounting for 54 patients. Osteosarcoma is the second most frequent diagnosis, with 14 cases, followed by chondrosarcoma and Ewing sarcoma, each with 7 cases. Less common diagnoses include dermatofibrosarcoma protuberans, with a single case, and giant cell tumor of bone, with two cases.

The dataset includes a total of 86 patients diagnosed with various sarcoma types and, since each patient underwent imaging in multiple anatomical planes, the study comprises 186 MRI exams. Although both T1- and T2-weighted sequences were included in the study to enhance robustness and generalizability, each patient was not necessarily represented by both modalities. These scans also include contrast-enhanced and non-enhanced images, as well as sequences acquired with and without fat suppression, thereby capturing a broad spectrum of real-world imaging conditions. The distribution of MRI exam types, including both contrast-enhanced and non-contrast sequences, is summarized in Table 1. None of the MRI sequence groups contains enough exams to be used individually; therefore, the sequences were aggregated to ensure a meaningful sample size, resulting in a total of 186 MRI exams.

In particular, the STIR case was not excluded since the tumor appearance is visually similar to that observed in T2 with Fat Saturation. Casale et al., in their study on metastasis prediction in soft tissue sarcoma cases using formal methods, employed both STIR and T2 images with fat saturation [17]. Due to the similarity between these sequences, including the STIR exam in our study does not introduce significant variability.

**Figure 1:**
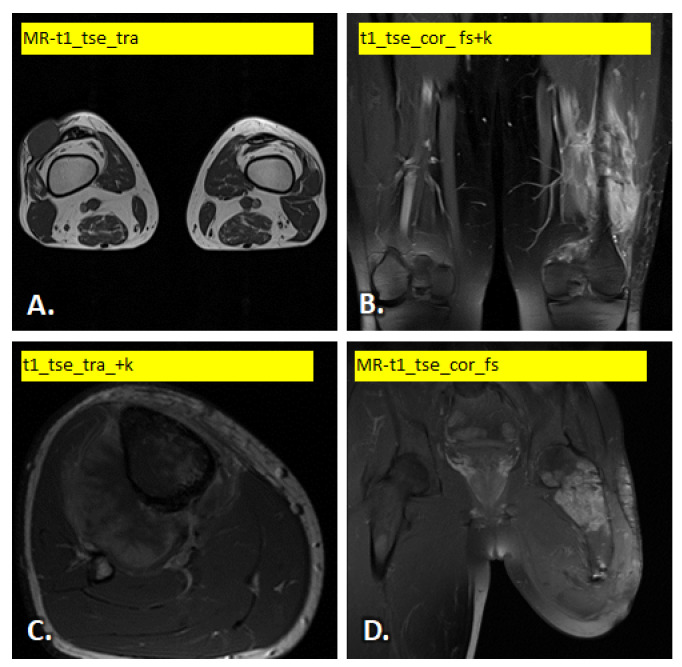
Example of T1-weighted MRI scan variants: (**A**) soft tissue tumor in the lateral joint capsule of the right knee, (**B**) bone tumor (Ewing) in the lower end of the left femur, (**C**) bone tumor on the proximal posterior side of the right tibia and (**D**) bone tumor in the trochanteric area of the left femur.

**Figure 2:**
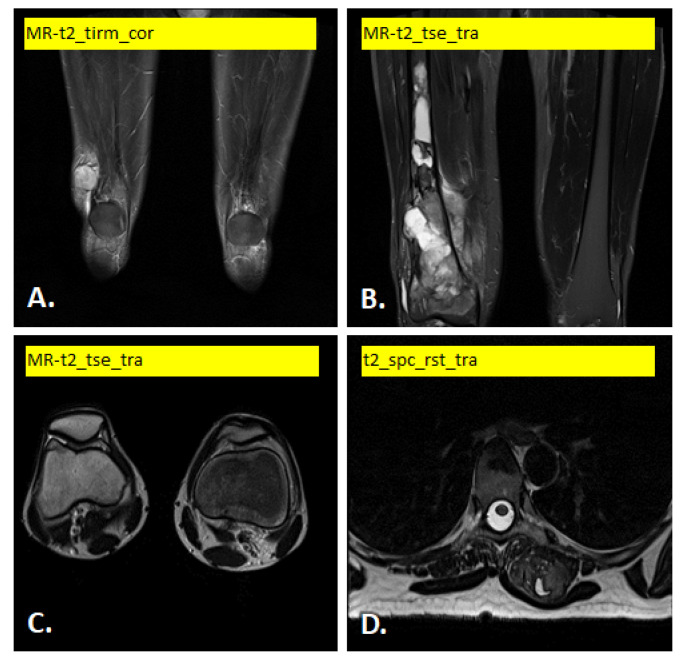
Example of T2-weighted MRI scan variants: (**A**) soft tissue tumor in lower end of right vastus lateralis muscle, (**B**) bone tumor in lower end of right femur, (**C**) soft tissue tumor in synovia of right knee joint and (**D**) soft tissue tumor in left erector spine muscle.

In Figure 1 and Figure 2 illustrate examples of the different images used.

#### 2.1.2. Healthy Cases

**Figure 3:**
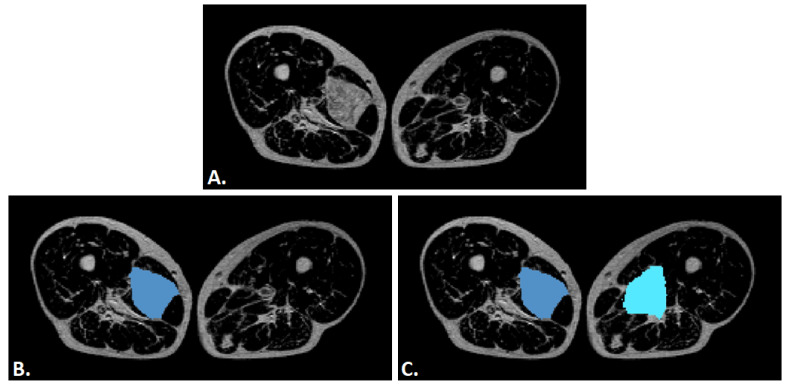
Example of tumor and healthy tissue segmentation in axial T1-weighted MRI of the thighs. (**A**) shows the original MRI with a unilateral soft tissue sarcoma. (**B**) displays the manual segmentation of the tumor (blue), while (**C**) shows the corresponding healthy tissue regions (light blue) from the contralateral limb, used as negative samples.

Although the dataset does not include scans from healthy subjects, regions of healthy tissue were extracted from the MRI scans of sarcoma patients, as illustrated in Figure 3. These regions were manually selected to represent non-pathological anatomy and were used to provide negative samples during model training. This method yielded 175 samples, contributing to a dataset balanced not only in terms of sample count. As a matter of fact, this approach ensures that both tumor and non-tumor tissue characteristics are learned within the same imaging context. The number of healthy tissue samples is moderately lower than that of sarcoma samples, as not all scans contained sufficient healthy tissue for safe segmentation. In such cases, the corresponding healthy images were excluded to prevent volumes from potentially containing tumor traces.

Figure 3A shows the original MRI image with a unilateral soft tissue sarcoma. Figure 3B displays the manual segmentation of the tumor region (blue) on the affected side, while Figure 3C illustrates the corresponding extraction of healthy tissue regions (light blue) from the contralateral limb. These were used as negative samples for training, in contrast to the tumor region (blue) used as the positive sample. This strategy enables intra-patient comparison under consistent imaging conditions.

### 2.2. Methods

**Figure 4:**
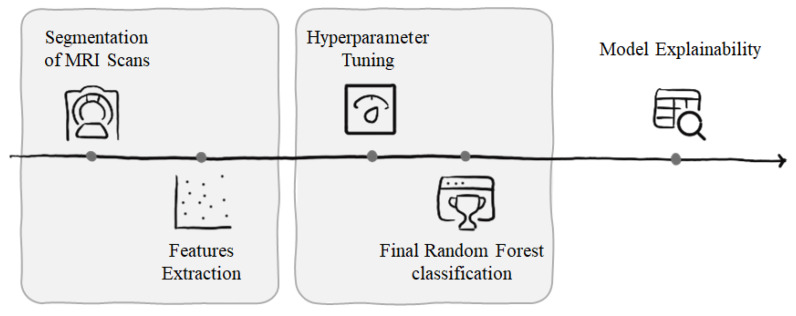
Workflow: segmentation of MRI scans, radiomic feature extraction, Random Forest classification after NCV, and model explainability using Feature Importance and LIME.

The workflow started with the segmentation of both sarcoma lesions and healthy tissue volumes from MRI scans. Radiomic features were then extracted from these segmented regions to quantitatively characterize tissue properties. A Random Forest classifier was employed for the classification task [18]. To optimize performance and reduce overfitting, a Nested Cross-Validation (NCV) strategy was adopted [19]. The inner loop was dedicated to hyperparameter tuning, while the outer loop provided an unbiased estimate of generalization performance. After identifying the optimal hyperparameters, the model was retrained on the entire training set. Finally, to improve model interpretability and support clinical decision-making, Feature Importance and Local Interpretable Model-agnostic Explanations (LIME) were applied to identify the most influential radiomic features for each prediction. The complete analysis pipeline is illustrated in Figure 4.

#### 2.2.1. Segmentation and Features Extraction

In this work, image segmentation was applied to identify and isolate specific regions within medical scans, resulting in the creation of masks. Segmented regions included the sarcoma tumor, labeled as “Sarcoma”, and, when present, healthy tissue, labeled as “Healthy”. As previously mentioned, healthy tissue samples were derived from the same sarcoma patient’s exams. Due to the absence of standardized scan orientation during acquisition, images in axial, coronal, and sagittal planes were included. Each available orientation for a patient was segmented, resulting in up to three image sets per individual. The segmentation process was carried out using Mimics v25.0 software (Materialize, Leuven, Belgium). A thorough visual inspection of MRI scans was performed to identify suitable regions for segmentation. Areas showing visible abnormalities (such as inflammation, edema, or other suspicious features) were carefully evaluated. For healthy tissue, regions showing signs such as inflammation, edema, or other suspicious features were excluded to ensure that the selected samples were as representative as possible of normal tissue within sarcoma patients. No control volumes were segmented when adequate and enough healthy tissue was not visible. Similarly, tumor regions were selected based on clear visual evidence of the lesion. All segmentations were manually performed and subsequently reviewed by an expert, but no formal inter-rater consistency assessment was conducted. Following segmentation, both MRI images and corresponding masks were exported for radiomic feature extraction and subsequent analysis. Features extraction from segmented volumes was carried out through PyRadiomics 3.1.0, a Python library compliant with the Image Biomarker Standardization Initiative (IBSI) [20]. Extracted feature groups span various categories, totaling 107 radiomic features. First-order statistical features describe the overall distribution of voxel intensities within the region of interest (e.g., mean, variance, skewness). Gray Level Co-occurrence Matrix (GLCM) features capture spatial relationships between pairs of pixels by assessing the frequency of specific gray-level combinations. Gray Level Size Zone Matrix (GLSZM) features quantify the size and distribution of homogeneous gray-level zones, extending beyond simple pixel pair comparisons. Gray Level Run Length Matrix (GLRLM) features evaluate the length of consecutive pixels with the same intensity along specific directions. Neighbouring Gray Tone Difference Matrix (NGTDM) features describe local intensity variations by measuring differences between a pixel and its surrounding neighbors, capturing aspects such as contrast and texture coarseness [21]. Shape3D features were included to characterize the geometric properties of the segmented volumes, such as surface area, compactness, sphericity, and elongation, offering insight into the overall morphology of the regions of interest [22].

Furthermore, wavelet transforms were applied to generate higher-order features by decomposing the original image into eight distinct frequency sub-bands, using combinations of low-pass (L) and high-pass (H) filters along the X, Y, and Z axes [23]. These components, listed in Table 2, include one approximation image (LLL), capturing low-frequency content and overall structural information, and seven detail images (e.g., LLH, HHL, HHH), which emphasize high-frequency patterns such as edges, fine textures, and directional details.

From each of these eight wavelet-derived volumes, 93 radiomic features were extracted, including first-order statistics and texture descriptors. Shape-based features were excluded from the wavelet-derived volumes, as they depend only on the spatial mask and not on gray-level intensities.

The original image contributed 107 features: 93 intensity-based and texture features, and 14 shape-based features. Applying the same 93 intensity-based features to each of the eight wavelet sub-bands yielded an additional 744 features (93 × 8). Combining all components, the final feature set used for classification comprised 851 features:

$$\mathrm{Total}\;\mathrm{features}=\underset{\mathrm{original}}{\underset{︸}{93+14}}+\underset{\mathrm{wavelet}}{\underset{︸}{93\times 8}}=851$$

All features were extracted following the PyRadiomics standard pipeline [20].

Manually extracted features were chosen instead of deep features because deep learning approaches typically require large-scale datasets, whereas handcrafted features are more suitable for scenarios with limited data availability, as is the case here [24,25,26].

#### 2.2.2. Hyperparameter Tuning and Random Forest Classification

Following image segmentation with Mimics Innovation Suite (Materialise NV) and radiomic feature extraction via PyRadiomics, hyperparameter tuning was conducted using Python 3.9. Key libraries included scikit-learn v1.5.1 for machine learning, pandas v2.2.1 for data manipulation, and numpy v1.26.4 for numerical operations commonly employed in AI healthcare applications [27,28,29].

Prior to model training, the dataset was partitioned using a stratified hold-out strategy. A test set of 58 samples was reserved as an independent hold-out subset, used exclusively for the final evaluation of model performance. The remaining samples constituted the development set, employed for model optimization and validation. This stratified partitioning preserved class distribution across both subsets, minimizing sampling bias and enhancing generalizability.

To ensure unbiased performance estimation and robust hyperparameter selection, we adopted a Nested Cross-Validation (NCV) framework. NCV is used for evaluating machine learning models when hyperparameters require tuning, as it effectively prevents information leakage between the model selection and evaluation phases. We implemented a *k*-fold stratified outer loop to assess generalization capability, coupled with a *k*-fold stratified inner loop for hyperparameter optimization. Stratification was used in both loops to maintain consistent class distributions across all folds.

Numerous studies report that k=10 and k=5 are the most commonly used values in *k*-fold cross-validation among machine learning practitioners. However, the literature highlights that there is no definitive rule for choosing the value of *k* [30]. In our case, we selected k=9 for the outer loop, as the more commonly used values did not yield a unique or consistent selection of model parameters during validation.

Within each outer loop iteration, a Grid Search was performed over a predefined hyperparameter set for the Random Forest classifier, including the number of estimators (n_estimators), maximum tree depth (max_depth), and the minimum number of samples required to split an internal node (min_samples_split). The parameter grid included:

- *n_estimators*: 100, 200, and 1000,
- *max_depth*: None, 5, and 10,
- *min_samples_split*: 2 and 5.

Tuning *max_depth* and *min_samples_split* helps control and mitigate overfitting by limiting the growth and complexity of individual trees, while adjusting *n_estimators* balances the trade-off between computational cost and model variance. Setting *max_depth* to None allows each decision tree in the forest to grow without a predefined limit, enabling the model to capture complex patterns in the data. Setting *min_samples_split* to 2 allows nodes to be split as long as they contain at least two samples, encouraging fine-grained partitioning. Finally, choosing 200 for *n_estimators* offers a practical trade-off between performance and computational efficiency.

For every outer fold and its corresponding best combination of hyperparameters, we evaluated model performance using the metrics: accuracy, precision, recall, F1-score, and the area under the ROC curve (ROC-AUC). Employing a diverse set of evaluation metrics provides a more comprehensive view of classifier behavior.

The final Random Forest model was retrained on the entire development set using the optimal hyperparameters identified through the NCV process. It was subsequently evaluated on the unseen hold-out test set to obtain an unbiased performance estimate. The test set comprised 28 Healthy patients and 30 Sarcoma patients.

#### 2.2.3. Model Explainability

To enhance the interpretability of the final Random Forest model, two explainability techniques were employed: Feature Importance and Local Interpretable Model-agnostic Explanations (LIME). These methods offer insights into global and local model behavior and help ensure transparency and trust in the model’s decisions, which is important in medical imaging.

Global interpretability was achieved by analyzing Feature Importance via the model’s *feature_importances_* attribute. This metric quantifies each feature’s contribution to the model’s predictions, based on the average decrease in impurity across all trees. Features were ranked according to their importance scores to identify the most relevant variables for classification. This analysis was conducted on the final Random Forest model, retrained on the full development set using optimal hyperparameters identified via nested cross-validation. The resulting importance scores were used to determine which radiomic features most influenced the model’s decision-making process.

LIME [31] was involved to explain individual predictions by identifying the most influential features and quantifying their impact. Each feature is associated with a value range and an assigned weight. A positive weight indicates that the feature supports the predicted class, while a negative weight suggests influence toward another class. This approach is particularly valuable when applied to complex, non-linear models like Random Forests, which comprise ensembles of decision trees and are inherently difficult to interpret. By approximating the local behavior of the Random Forest with a simpler, interpretable model, LIME enables users to understand which features contributed most to a specific prediction. This enhances transparency and trust, particularly in domains where interpretability is critical, such as medicine. In particular, LIME is introduced for its ability to provide interpretable and accurate explanations for the outputs of any classification model. LIME was implemented using the *lime* Python package, specifically the *lime.lime_tabular.LimeTabularExplainer* class.

## 3. Results

### 3.1. Results of the Hyperparameter Tuning

Table 3 presents the optimal hyperparameter combinations and corresponding performance metrics for each outer fold. Despite variations across folds, the most frequently selected configuration was max_depth = None, min_samples_split = 2, n_estimators = 200.

### 3.2. Final Random Forest Classification

**Figure 5:**
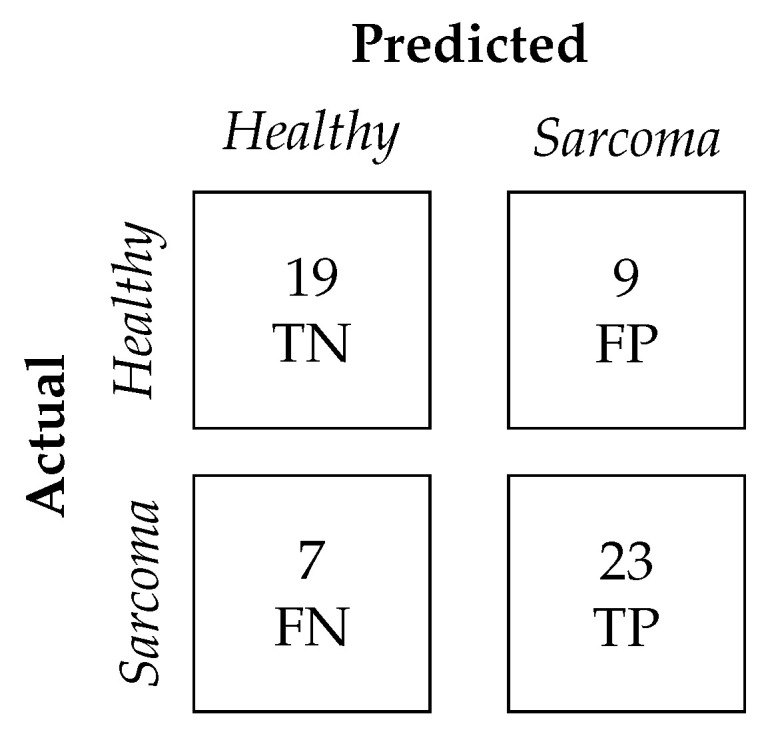
Confusion matrix for classification on the hold-out test set.

The resulting confusion matrix, shown in Figure 5, illustrates the classification outcomes. In the matrix, TN (True Negatives) denotes Healthy samples correctly classified as Healthy, while FP (False Positives) represents Healthy samples incorrectly classified as Sarcoma. FN (False Negatives) corresponds to Sarcoma cases misclassified as Healthy, while TP (True Positives) refers to Sarcoma samples correctly identified.

TPerformance metrics obtained from the hold-out test set are presented in Table 4. This approach ensured that the metrics were not biased by the model selection process.

### 3.3. Model Explainability

#### 3.3.1. Feature Importance Results

As a result of the analysis, 849 out of 851 features contributed to the model’s decisions, even if only marginally. Table 5 lists the ten most influential features used in the classification.

Feature importance analysis indicated that a broad spectrum of radiomic features contributed to the model’s decision-making process, with the highest-ranked features predominantly derived from wavelet-transformed images, especially from the LHL and HLL decomposition levels. This suggests that texture patterns captured through wavelet filtering play a crucial role in distinguishing between healthy and sarcoma tissues. Many of the most influential features were first-order statistical descriptors (e.g., mean, median, skewness), indicating that the distribution of voxel intensities within the segmented regions carries significant discriminative information. Additionally, texture-based features derived from GLDM, GLRLM, and GLCM were also prominent. These features reflect spatial relationships and structural complexity within the tissue, such as the presence of fine textures, low gray-level emphasis, and local intensity variations. The integration of intensity-based and texture-based descriptors, especially those extracted from wavelet domains, highlights the model’s reliance on both global and localized image characteristics. This diverse feature set aligns with the heterogeneous nature of sarcoma, which often exhibits complex and subtle imaging patterns. Overall, the feature importance analysis reinforces the biological plausibility of the model’s predictions and strengthens confidence in its interpretability.

#### 3.3.2. Local Interpretability with LIME

To illustrate the behavior of the model across different outcomes, LIME was applied to four representative test cases, covering all combinations of ground truth and predicted labels (where 0 refers to “Healthy” and 1 to “Sarcoma”):

- Case 1: true = 0, predicted = 0 (TN);
- Case 2: true = 1, predicted = 1 (TP);
- Case 3: true = 0, predicted = 1 (FP);
- Case 4: true = 1, predicted = 0 (FN).

It is worth noting that individual feature contributions were relatively small (mostly in the range of ±0.01 to ±0.03), which is expected given that the model’s decisions are based on a high-dimensional feature space comprising 849 radiomic features. In such high-dimensional settings, the overall decision is distributed across numerous features, with no single attribute dominating the prediction.

**Figure 6:**
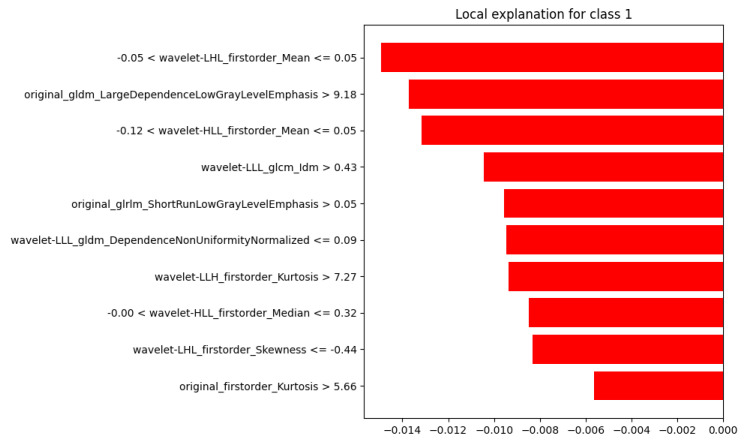
LIME Explanation: True =0, Predicted =0 (Correct Classification).

Case 1, illustrated in Figure 6, showed that all ten top-ranked features had negative contributions, consistently driving the prediction toward class 0.

**Figure 7:**
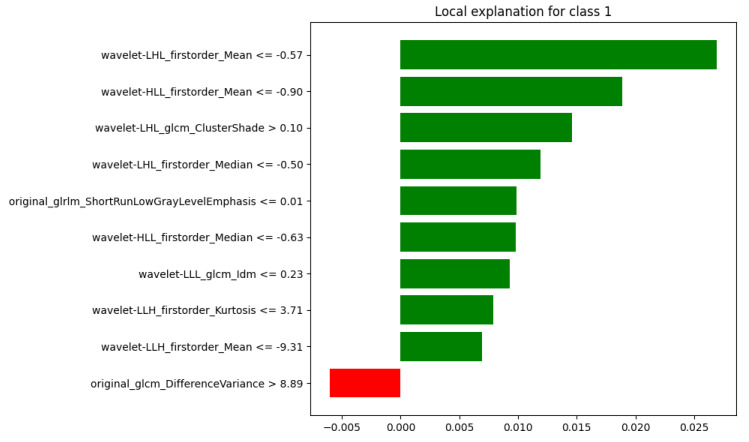
LIME Explanation: True =1, Predicted =1 (Correct Classification).

In Case 2, reported in Figure 7, the outcome was strongly supported by the features: nine out of ten exhibited positive contributions promoting class 1, while only one showed a small negative weight. This indicates a confident and coherent decision aligned with the true label.

**Figure 8:**
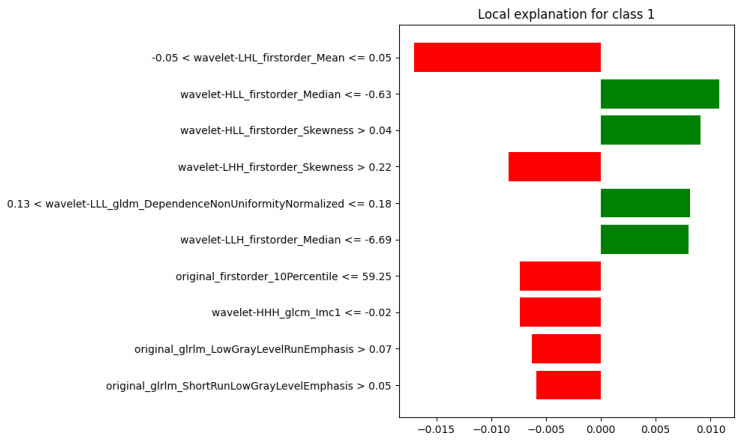
LIME Explanation: True =0, Predicted =1 (Misclassification).

Case 3, depicted in Figure 8, corresponded to a misclassification. Here, the contributions were mixed, with some features supporting the true label through negative weights, and others reinforcing the incorrect prediction with positive ones. The stronger effect of the latter was sufficient to overturn the decision, highlighting how small variations in specific features can lead to errors.

**Figure 9:**
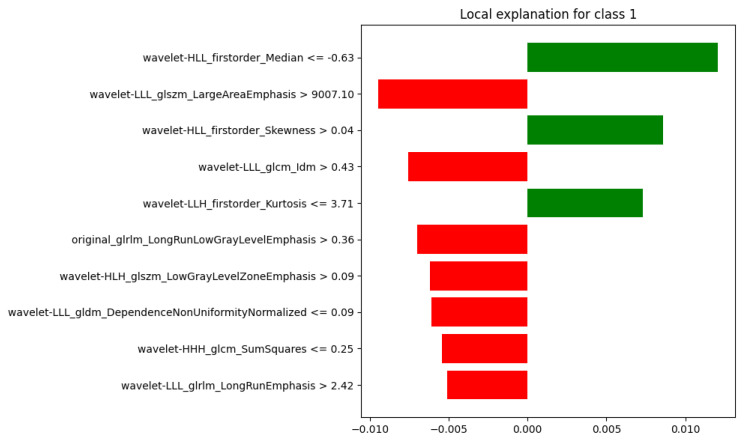
LIME Explanation: True =1, Predicted =0 (Misclassification).

Finally, Case 4, shown in Figure 9, represented another misclassification. Most influential features had negative contributions pulling the prediction toward class 0, while only three provided weak support for class 1. The model thus behaved consistently with the observed feature values, but the decision was ultimately incorrect, possibly reflecting overlapping feature distributions between the two classes.

### 3.4. Performance Comparison

Table 6 presents a comparative evaluation of several classification algorithms applied to the same dataset used by the proposed method. Models were evaluated using five standard metrics: Accuracy, Precision, Recall, F1-score, and ROC-AUC, in order to benchmark their performance against that of the Random Forest classifier. All classifications were performed using WEKA (Version 3.8.6, Hamilton, New Zealand), a software platform designed for machine learning experimentation and data analysis.

LWL and SVM showed competitive results, especially in Accuracy and F1-score. However, the proposed method based on Random Forest, achieved the best overall performance, obtaining the highest F1-score (0.742) and ROC-AUC (0.871), achieving the highest F1-score (0.742) and ROC-AUC (0.871), which reflect strong class discrimination and a well-balanced trade-off between precision and recall. Although IBK achieved a relatively high Recall (0.741), it had lower Precision (0.571). Naive Bayes and BayesNet exhibited a tendency to overpredict the positive class, leading to imbalanced performance across metrics. KStar, in particular, consistently assigned all patients to the negative class, failing to correctly identify any positive cases. Consequently, Precision and F1-score could not be computed, and its ROC-AUC value remained close to chance level (0.500), highlighting its poor discriminatory ability for this classification task.

## 4. Discussion

In this work, we investigated the effectivenessof machine learning techniques for sarcoma diagnosis. Specifically, classification was performed using a Random Forest model on radiomic features extracted from manually segmented volumes containing either sarcoma or healthy tissue.

The real strength of this study lies in the results obtained using heterogeneous MRI images acquired with varying protocols, both with and without contrast, and from multiple anatomical regions. Additionally, the dataset spans a wide temporal range (2004–2023), capturing significant variability in MRI technology and acquisition parameters. The sample size of 86 patients is a limitation and a constraint in terms of the generalizability of the results. For this very reason, examinations of different plans were used for each patient, thus obtaining 186 “Sarcoma” samples for classification. Future work will focus on validating the model on larger datasets to improve robustness and generalizability.

Due to this high heterogeneity, the classification task was particularly challenging and required the use of 849 out of 851 available radiomic features to achieve robust performance. This underscores the generalizability of our approach across diverse imaging conditions, which is a critical requirement for real-world clinical applications. These findings are especially encouraging considering the complexity of the task, which involves differentiating between pathological and non-pathological tissue across a heterogeneous set of MRI acquisitions. The dataset includes various sarcoma types and imaging protocols, introducing significant variability in terms of anatomical location, acquisition parameters, and lesion appearance. Despite these challenges, the model maintained robust and consistent predictive performance, further supporting its potential clinical applicability. Assessing the contribution of individual MRI sequences could potentially enhance classification performance. However, conducting separate analyses for each modality would have resulted in a further reduction of the effective sample size, due to the incomplete availability of all sequences for every patient. Given the already limited dataset, such stratification would have compromised statistical power and model reliability. For this reason, we opted to combine available sequences to maximize the use of data and ensure more stable model training. Furthermore, the application of explainability techniques provided valuable insights into the model’s decision-making process. These explainability analyses highlighted specific radiomic features, particularly wavelet-transformed first-order and texture metrics, as influential predictors, enhancing the model’s transparency and potential clinical trustworthiness.

This suggests that texture patterns captured via wavelet filtering play a crucial role in distinguishing between healthy and sarcoma tissues. Several of the most influential features were first-order statistics (such as mean, median, and skewness), indicating that the distribution of voxel intensities within the segmented regions carries significant discriminative information. Additionally, texture-based features derived from GLDM, GLRLM, and GLCM were also prominent. These features reflect spatial relationships and structural complexity within the tissue, such as the presence of fine textures, low gray-level emphasis, and local intensity variations. The combination of intensity-based and texture-based descriptors, especially those extracted from wavelet domains, highlights the model’s reliance on both global and localized image characteristics. This diverse feature set aligns with the heterogeneous nature of sarcoma, which often exhibits complex and subtle imaging patterns. Overall, the feature importance analysis supports the biological plausibility of the model’s predictions and enhances confidence in its interpretability.

Prioritizing the F1-score is especially justified in medical domain, where both false positives and false negatives can have significant clinical consequences. The F1-score, as the harmonic mean of precision and recall, provides a balanced measure that accounts for both types of errors. In healthcare applications, an overemphasis on either precision or recall alone may lead to suboptimal patient outcomes, such as missing true cases (low recall) or causing unnecessary interventions (low precision). By maximizing the F1-score, we aim to achieve a clinically meaningful balance between sensitivity and specificity, ultimately supporting safer and more effective decision-making.

This approach ensures that performance metrics accurately reflect the model’s generalization capability, minimizing the risk of overly optimistic estimates due to selection bias or overfitting.

It is important to note that individual feature contributions were relatively small (mostly in the range of ±0.01 to ±0.03), which is expected given that the model’s decisions are based on a high-dimensional feature space comprising 849 radiomic features. In such cases, the overall decision is distributed across many features, with no single attribute dominating the prediction.

Nevertheless, recent advances in machine learning, particularly deep learning, have significantly enhanced the analysis of radiological images for the diagnosis and management of sarcomas as well as the quantification of the pathologists’ visual pattern for whole slide image diagnosis [32].

Our research group recently explored the use of artificial intelligence for for sarcoma diagnosis and grading based on MRI scans and radiomics [33]. In particular, when focused on the binary classification (i.e., diagnosis of sarcoma), they achieved an accuracy of 76.02%. Although starting from the same dataset, the main difference from this study is that the authors analyzed individual slices, thus obtaining a larger dataset, whereas in the present work, volumes were considered in their entirety. In addition, segmentation of healthy tissue was performed by excluding areas containing sarcoma and edema and including the rest of the image. From a methodological standpoint, although Random Forest was also employed in that study, validation was conducted by leave-one-out cross-validation. By contrast, in the study presented here, a portion of the dataset was isolated from the beginning and used exclusively for final validation. The remaining dataset was used for model tuning, performed through nested k-fold cross-validation, and subsequently for final training, in order to avoid any form of data leakage.

By contrast, several studies have focused on lipomas, which are benign fatty tumors. For example, Gitto et al. analyzed 150 cases to differentiate lipomas from atypical (low-grade sarcomas) lipomatous tumors using three models. Their best model achieved 92% sensitivity but only 33% specificity, suggesting a high false positive rate likely due to dataset imbalance [34]. In contrast, Malinauskaite et al. evaluated 38 cases to distinguish lipomas from liposarcomas using four models. The Support Vector Machine (SVM) performed best, achieving an AUC of 0.926, 88% sensitivity, perfect specificity (100), and 92.7% accuracy [35]. Unlike these studies, our work is based on a larger and balanced dataset comprising 361 patients (186 with sarcoma and 175 healthy), allowing for more robust and generalizable model training and evaluation.

The study by Dai et al. involved 172 patients and evaluated four different CNN architectures for deep feature extraction, combined with a Random Forest classifier, to distinguish uterine sarcomas from atypical leiomyomas [36]. In their approach, the authors based the analysis on only three MRI slices, whereas our method leverages the entire tumor volume, providing a more comprehensive representation of the lesion.

Collectively, these studies demonstrate that machine learning techniques can enhance diagnostic accuracy and patient stratification in soft tissue tumors. However, their performance varies depending on the model used, sample size, and complexity of the diagnostic task. In fact, the main difference between our work and the cited studies is that we distinguish between healthy tissue and sarcomas, encompassing nearly all types rather than focusing on a specific niche.

Although developed in a different domain, the approach proposed by Nan et al. [32] uses expert-derived information in the form of pathologists’ gaze patterns to train deep learning models for histopathological diagnosis. In contrast, our model relies on structured clinical data and confirmed diagnoses to produce interpretable predictions at the time of referral. These represent complementary strategies for integrating clinical expertise into decision-support systems.

Researchers are also exploring Generative Adversarial Networks (GANs) to generate synthetic radiological images, addressing the challenge of limited training data due to the rarity of sarcomas [37,38]. A promising research direction involves integrating radiological features with other data modalities (such as histopathological, transcriptomic, and genomic data) through multi-modal AI systems [39,40,41].

Despite the encouraging results, limitations including the relatively small dataset size, variability in imaging planes, and manual segmentation processes suggest the need for further validation on larger, multi-center cohorts and the investigation of automated segmentation methods. Moreover, the “black box” nature of deep learning models poses interpretability challenges that may hinder clinical adoption. However, as demonstrated in this study, Explainable AI techniques are being explored to improve model transparency and foster trust in AI-driven decisions.

In conclusion, although technical and logistical barriers persist, machine learning is poised to transform radiological practice in sarcoma care, offering the potential for earlier diagnosis, more accurate prognostication, and truly personalized therapy.

## 5. Conclusions

This study demonstrates the feasibility of using radiomic features from MRI combined with a Random Forest classifier to distinguish sarcoma from healthy tissue:

1. The model, optimized via nested cross-validation, achieved promising performance on an independent test set (F1-score: 0.742), showing a balanced trade-off between sensitivity and specificity.
2. The integration of explainability techniques, including global feature importance and LIME, offered valuable insights into the model’s decision-making process, enhancing its transparency and clinical relevance. These findings underscore the potential of radiomics and machine learning as impactful tools in oncologic imaging.

Future work must focus on integrating multimodal imaging data and advanced deep learning architectures to further enhance classification accuracy and model robustness.

## Figures and Tables

**Table 1:** Number of MRI exams by sequence type, combining contrast-enhanced variants.

Type	# Exams T1	# Exams T2
No Fat Saturation and No Contrast	31	54
With Fat Saturation	13	25
With Fat Saturation and Contrast	29	0
With Contrast	19	0
STIR	1	
Total	95	91

**Table 2:** 3D Wavelet Transform Components.

Combination	Interpretation
LLL	Low-pass on X, Y, and Z (global structure)
LLH	Low-pass on X and Y, High-pass on Z
LHL	Low-pass on X and Z, High-pass on Y
LHH	Low-pass on X, High-pass on Y and Z
HLL	High-pass on X, Low-pass on Y and Z
HLH	High-pass on X and Z, Low-pass on Y
HHL	High-pass on X and Y, Low-pass on Z
HHH	High-pass on X, Y, and Z (fine details)

**Table 3:** Hyperparameter combinations and performance metrics across outer folds.

Outer Fold	Best Combinations	Accuracy	Precision	Recall	F1-Score	ROC-AUC
1	max_depth: None	0.765	0.812	0.722	0.765	0.885
	min_samples_split: 2					
	n_estimators: 200					
2	max_depth: None	0.794	0.867	0.722	0.788	0.872
	min_samples_split: 2					
	n_estimators: 200					
3	max_depth: 10	0.794	0.789	0.833	0.811	0.878
	min_samples_split: 2					
	n_estimators: 200					
4	max_depth: 5	0.765	0.765	0.765	0.765	0.827
	min_samples_split: 5					
	n_estimators: 1000					
5	max_depth: None	0.853	0.875	0.824	0.848	0.938
	min_samples_split: 2					
	n_estimators: 1000					
6	max_depth: 10	0.727	0.700	0.824	0.757	0.768
	min_samples_split: 5					
	n_estimators: 200					
7	max_depth: 5	0.818	0.762	0.941	0.842	0.886
	min_samples_split: 2					
	n_estimators: 1000					
8	max_depth: None	0.758	0.714	0.882	0.789	0.831
	min_samples_split: 5					
	n_estimators: 200					
9	max_depth: 10	0.758	0.714	0.882	0.789	0.860
	min_samples_split: 5					
	n_estimators: 100					

**Table 4:** Performance metrics from classification on the hold-out test set.

Accuracy	Precision	Recall	F1-Score	ROC-AUC
0.724	0.719	0.767	0.742	0.871

**Table 5:** Top 10 most important features ranked by Random Forest.

Feature	Importance
wavelet-LHL_firstorder_Mean	0.024852
wavelet-HLL_firstorder_Median	0.015829
wavelet-HLL_firstorder_Mean	0.015710
original_gldm_LargeDependenceLowGrayLevelEmphasis	0.013242
wavelet-LHL_firstorder_Median	0.011875
wavelet-LHL_glcm_ClusterShade	0.010387
original_gldm_LowGrayLevelEmphasis	0.010175
original_glrlm_LowGrayLevelRunEmphasis	0.009607
original_glrlm_ShortRunLowGrayLevelEmphasis	0.009075
wavelet-HLL_firstorder_Skewness	0.009016

**Table 6:** Comparative performance of classification algorithms on the balanced binary dataset.

Classifier	Accuracy	Precision	Recall	F1-Score	ROC-AUC
RandomForest	0.724	0.719	0.767	0.742	0.871
RandomTree	0.706	0.593	0.593	0.653	0.700
IBK	0.529	0.571	0.741	0.645	0.628
LWL	0.741	0.773	0.630	0.694	0.740
KStar	0.534		0.000		0.500
SVM	0.724	0.739	0.630	0.680	0.718
NaiveBayes	0.573	0.475	0.704	0.567	0.512
BayesNet	0.517	0.487	0.704	0.576	0.552
AdaBoostM1	0.672	0.654	0.630	0.642	0.781

## Data Availability

The data are not publicly available but can be obtained from the corresponding author upon reasonable request.
